# Coloc-stats: a unified web interface to perform colocalization analysis of genomic features

**DOI:** 10.1093/nar/gky474

**Published:** 2018-06-05

**Authors:** Boris Simovski, Chakravarthi Kanduri, Sveinung Gundersen, Dmytro Titov, Diana Domanska, Christoph Bock, Lara Bossini-Castillo, Maria Chikina, Alexander Favorov, Ryan M Layer, Andrey A Mironov, Aaron R Quinlan, Nathan C Sheffield, Gosia Trynka, Geir K Sandve

**Affiliations:** 1 Department of Informatics, University of Oslo, Gaustadalléen 23 B, N-0373 Oslo, Norway; 2 K. G. Jebsen Centre for Coeliac Disease Research, Oslo University Hospital, Sognsvannsveien 20, 0372 Oslo, Norway; 3 Elixir Norway - Oslo node, Department of Informatics, University of Oslo, Gaustadalléen 23 B, N-0373 Oslo, Norway; 4 CeMM Research Center for Molecular Medicine of the Austrian Academy of Sciences, 1090 Vienna, Austria; 5 Department of Laboratory Medicine, Medical University of Vienna, 1090 Vienna, Austria; 6 Max Planck Institute for Informatics, 66123 Saarbrücken, Germany; 7 Cellular Genetics Programme, Wellcome Sanger Institute, CB10 1SA Hinxton, UK; 8 University of Pittsburgh School of Medicine, 3550 Terrace Street, Pittsburgh, PA 15213, USA; 9 Department of Oncology, Sidney Kimmel Comprehensive Cancer Center, The Johns Hopkins University School of Medicine, 550 N Broadway, Baltimore, MD 21205, USA; 10 Laboratory of Systems Biology and Computational Genetics, Vavilov Institute of General Genetics, Gubkina Street 3, Moscow 119333, Russia; 11 Department of Human Genetics, University of Utah, 15 N 2030 E, Salt Lake City, UT 84112, USA; 12 USTAR Center for Genetic Discovery, University of Utah, 15 N 2030 E, Salt Lake City, UT 84112, USA; 13 Faculty of Bioengineering and Bioinformatics, Lomonosov Moscow State University, Lab. Bldg B, Vorobiovy Gory 1-73, Moscow 119992, Russia; 14 Skolkovo Institute of Science and Technology, Nobelya ul. 3, Moscow 121205, Russia; 15 Institute for Information Transmission Problems, Russian Academy of Sciences, Bolshoi Karenty per. 19, Moscow 127994, Russia; 16 Department of Biomedical Informatics, University of Utah, 421 Wakara Way, Salt Lake City, UT 84108, USA; 17 Center for Public Health Genomics, University of Virginia, Charlottesville, VA 22903 USA

## Abstract

Functional genomics assays produce sets of genomic regions as one of their main outputs. To biologically interpret such region-sets, researchers often use colocalization analysis, where the statistical significance of colocalization (overlap, spatial proximity) between two or more region-sets is tested. Existing colocalization analysis tools vary in the statistical methodology and analysis approaches, thus potentially providing different conclusions for the same research question. As the findings of colocalization analysis are often the basis for follow-up experiments, it is helpful to use several tools in parallel and to compare the results. We developed the Coloc-stats web service to facilitate such analyses. Coloc-stats provides a unified interface to perform colocalization analysis across various analytical methods and method-specific options (e.g. colocalization measures, resolution, null models). Coloc-stats helps the user to find a method that supports their experimental requirements and allows for a straightforward comparison across methods. Coloc-stats is implemented as a web server with a graphical user interface that assists users with configuring their colocalization analyses. Coloc-stats is freely available at https://hyperbrowser.uio.no/coloc-stats/.

## INTRODUCTION

High-throughput sequencing methods assay various genomic and epigenomic features, including regulatory elements, transcription factor binding sites (TFBS) and transcribed regions (1,2). Functionally related genomic features often co-occur within a genomic sequence [e.g. co-occurrence of TFBS (3)]. One important way of determining whether genomic features are functionally related to search for significant colocalization (based on overlap or proximity). The methodology that determines the significance of colocalization of genomic features is often referred to as colocalization analysis or as co-occurrence analysis or region set enrichment analysis. In a typical colocalization analysis, arithmetic set operations are performed between genomic tracks to determine the amount of overlap (overlapping sequence nucleotides) or spatial proximity (e.g. geometric distance between genomic regions). Statistical tests determine whether the observed overlap or spatial proximity is likely due to chance. Several tools have been developed with diverse functionalities to perform statistical testing of colocalization between a pair of tracks [e.g. (4–8)] or between multiple tracks [e.g. (9–13)].

**Figure 1.**
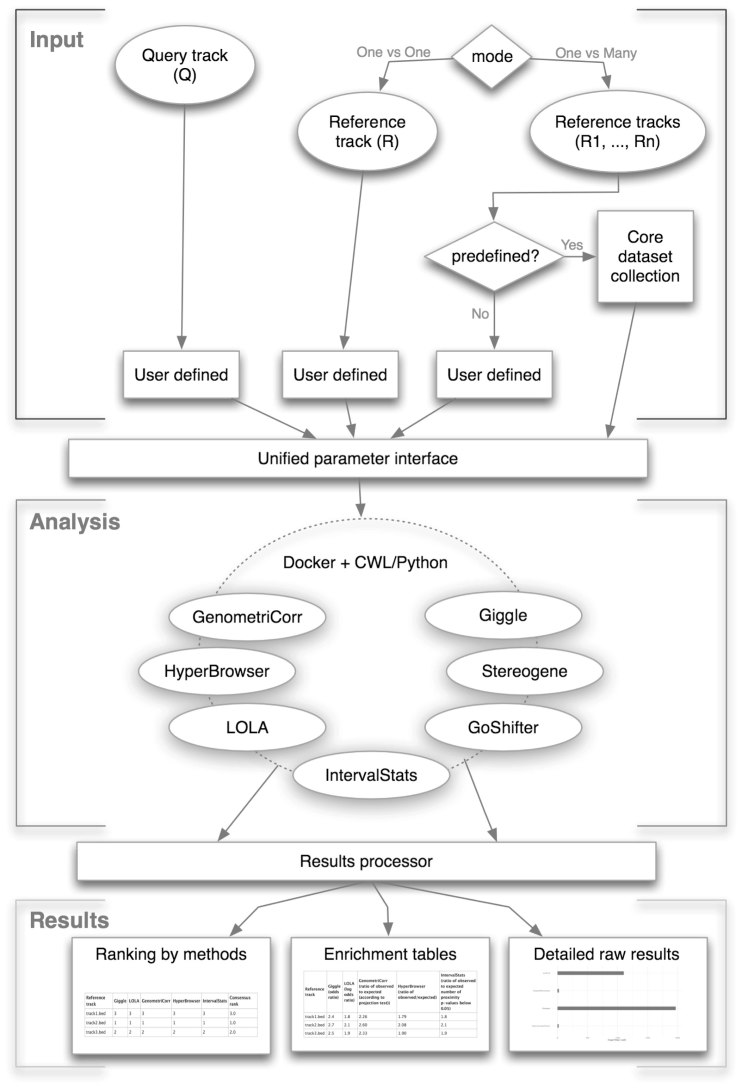
A schematic view of the Coloc-stats workflow. The input data can be either two tracks (query and reference), or one query track and a collection of reference tracks. The uniform interface mediates between the user’s selection parameters and the tool specific run configurations. The analysis layer contains all the tools and employs Docker and CWL to execute each tool with the appropriate parameter configurations. The results layer is responsible for collecting and presenting results in the form of rankings, descriptive statistics and *P*-value tables, alongside the raw output of each tool.

Despite the existence of several colocalization analysis tools, some important technical issues remain unaddressed. The available colocalization analysis methods use different concepts and null models to assess the significance of colocalization, and the choice of null models is known to affect the subsequent conclusions (14,15). Different parameter choices of the tools further increase the variation in conclusions. One way to overcome this uncertainty is to assess the consistency of the conclusions across different null models and parameter choices (14,15). However, there exists no single command-line or web-based tool that provides an easy and accessible unified interface to explore different colocalization analysis methods and examine the robustness of the findings. Also, some of the colocalization analysis tools that have unique and specialized functionalities are available only as command-line tools, which are less accessible to a substantial proportion of the scientific community who rely on web servers for most of their bioinformatics needs. To address these needs, we developed the Coloc-stats web server that provides a unified interface to multiple published methods of colocalization analysis. Figure 1 shows a schematic of the execution flow of Coloc-stats web server. Currently, seven different colocalization analysis tools are integrated in the web server, namely the Genomic HyperBrowser & GSuite HyperBrowser (4,10), GenometriCorr (5), IntervalStats (6), GoShifter (8), LOLA (9), Stereogene (11) and GIGGLE (13). The system is furthermore based on a modular design that allows future methods to be easily added. The Coloc-stats web server provides a significant enhancement of features over the existing individual tools by allowing users to:

- Explore and use multiple colocalization analysis tools in a single graphical user interface.
- Become aware of and consciously select between alternative modeling assumptions in order to arrive at a list of methods appropriate for their analysis scenario.
- Examine the robustness of conclusions by comparing results across several methods.
- Easily apply methods originally focused on pairwise relations to the analysis of entire track collections.

## FUNCTIONS AND FEATURES

### Multiple colocalization analysis tools integrated in a single web interface

To allow the users to explore and use multiple colocalization analysis methods in an easily accessible web interface, we have integrated seven different tools that have the generic aim of determining the significance of colocalization of genomic features. Since each of the integrated tools has specialized functionalities and unique analysis approaches, this integrated system serves as a comprehensive one-stop shop for performing colocalization analysis with wide range of approaches and functionalities. Below is a brief overview of the integrated tools and their unique and specialized analysis approaches.

- **GenometriCorr** determines the correlation of genomewide datasets. The R library implements statistical methods specific to the analysis of spatial correlation of genomic data. GenometriCorr makes use of Monte Carlo simulation to estimate the *P*-values and implements four different statistical tests on various relations between genomic tracks.
- **The Genomic HyperBrowser** performs statistical colocalization analysis in a way that allows null models and colocalization measures to be chosen independently and from a broad set of choices. Depending on biological assumptions chosen by the user, the statistical testing will be performed either by analytical approaches or Monte Carlo simulations, with a main emphasis on the later, as a more robust method. The methodology was, in the form of **GSuite HyperBrowser**, extended to support integrative analysis of track collections, with the Forbes coefficient selected as the default test statistic. The set of tools are provided through a user friendly web interface.
- **GIGGLE** searches an input file against all annotation files in a database and ranks the results based on a GIGGLE score. The command line accessible tool combines a Fisher’s Exact Test and the odds ratio of a 2×2 contingency table containing the number of intervals that are in (i) both the input and database files, (ii) solely the input file, (iii) solely the database file and (iv) neither the input file nor the database file (estimated by the difference between the union of the two sets and the quotient of the mean interval size of both sets and the genome size) to produce the score.
- **GoShifter** calculates the percentage of loci for which associated variants overlap with tested annotation and compares the observed value to permuted overlap derived by randomly shuffling annotations. The shuffles are independent across the loci and preserve both the linkage disequilibrium (LD) structure between the associated variants and the distance between the annotations. This ensures that the same number of variants and the annotation density at each locus are maintained with each permutation round. GoShifter is provided as a command line tool.
- **IntervalStats** is a command line tool that computes *P*-values for proximity of genomic elements, avoiding non-biological variation (like peak width) in the genomic datasets. Evaluation of similarity can be done on a restricted set of genomic regions instead of the whole reference genome.
- **LOLA** counts the number of overlapping regions between a user’s input query and various sets of genomic region contained in a reference database. It then does the same counting for a background (‘universe’) region set. The count numbers for the user and background overlaps of each region-set in the reference database are used to build a contingency table, and a Fisher’s exact test is used to assess the significance of the overlap, which is also quantified by the odds ratio. LOLA’s default reference database is derived from a variety of public data resources and can be extended or replaced by custom region-sets. LOLA is provided as an R library.
- **Stereogene** is a command line tool that computes spatial correlation of genomic datasets. It supports continuous signal data as well as the more common interval data. Through kernel correlation it captures the colocalization of genomic elements that need not overlap, but are close to each other.

### Conscious selection of an appropriate method compatible with analysis assumptions

Coloc-stats can be run either in a basic mode or in an advanced mode. The basic mode does not require the user to be familiar with all the analysis choices; instead, it runs with a set of default settings that are shared between all of the tools. The advanced mode provides more customization opportunities, allowing users to find and apply methods compatible with their analysis assumptions. The user can also select multiple alternatives for a given setting and get results from the same tool run multiple times with different settings. This will allow the user to become aware of and consciously select between alternative modeling assumptions in order to arrive at a list of methods appropriate for their analysis scenario.

### Extension of methods that support only a pair of tracks to support track collections

Most colocalization analysis tools compare a query track to either a single reference track (pairwise analysis) or to a collection of reference tracks (one-against-many analysis). The typical use cases of one-against-many analysis are often centered on the ranking of the reference tracks based on the degree of their colocalization enrichment with the query track. Thus, tools that innately support one-against-many analysis mainly report the ranks of reference tracks alongside the *P*-values, colocalization enrichment statistics and other derived statistics. Notably, the rankings of reference tracks reported by different tools may not necessarily agree because of the usage of different null models and parameter choices. Therefore, coloc-stats provides a functionality to obtain a consensus ranking for each reference track across different tools and null models. For this coloc-stats extends the pairwise analysis tools to handle one-against-many analysis, where the core statistical methodology of the individual tools remain unmodified. The resulting colocalization enrichment statistics are used to rank the reference tracks, which are subsequently used to obtain a consensus rank for each track as the geometric mean of all individual ranks.

### Assessment of consistency and complementarity of conclusions

Assessing the consistency of colocalization analysis conclusions across alternative analytical approaches is a preferable way to avoid false-positives (14,15). Although individual tools offer some degree of customization, the broadest variability in analytical approach is found between different methods. However, performing an analysis using multiple tools currently requires to install each individual tool, learn the specific functioning of each tool, prepare the data in a format compatible with each tool and find the relevant results in the different forms of output provided by each tool.

To fill these gaps, Coloc-stats provides a unified system of parameter selection, data preparation and result collection across multiple tools. It allows to easily run several tools and parameter variations simultaneously so that the consistency of conclusions can be examined. In the basic mode, each tool is run with its own default values, allowing beginner users to assess consistency of results according to a variety of approaches at a low technical threshold. The advanced mode allows more experienced users to systematically assess how results vary according to variation of particular parameters, as well as assessing the remaining variability of results across tools after fixing the value of certain parameters. The advanced mode provides a unified specification of the definition of colocalization (direct overlap or proximity), resolution of analysis (single basepair or broader regions), whether to preserve genomic structure and clumping tendencies (heterogeneity in feature occurrence along the genome) and whether to restrict the analysis to certain regions (by providing a set of universe regions or a set of regions to be excluded from analysis).

This way of running multiple colocalization analysis methods would result in multiple findings that may or may not necessarily agree with each other. In either case, we generally advice to report all *P*-values. If only *P*-values for certain methods/parameterizations are to be reported, this should be based on explicit reasoning in terms of which analytical assumptions are reasonable, not based on which *P*-values are desirable in themselves (a practice known as *P*-value hacking).

### Getting started with Coloc-stats

The web page of Coloc-stats contains a variety of material to help users get started with the web tool:

- A screencast of how to perform a basic analysis.
- A Galaxy history containing an example analysis, where results and parameter selections can be inspected and the analysis redone in its original or in a modified form.
- Sample data that allows to quickly try out the tool.
- A rich documentation that includes a detailed explanation of parameters and input and output data, as well as a FAQ that covers a variety of potential questions regarding usage of the web system.
- The tool itself includes help text for all selections in the GUI, and the results pages include brief guidance on how to interpret the results.

There are two main scenarios in which colocalization analysis is applied: (A) to determine whether or not two tracks (a query and a reference track) show a statistically significant degree of colocalization, and (B) to rank a set of reference tracks based on their degree of colocalization with a query track of interest. In scenario A one would supply a query and reference tracks to a Coloc-stats run and get back detailed results regarding the relation between these two tracks. In scenario B one would supply a query track and a set of reference tracks, and get back results indicating which of the reference tracks were colocalizing most strongly with the query track.

Regardless of scenario, performing an analysis with Coloc-stats consists of three steps:

- **Get datasets**, either:
  - Upload query and reference tracks (bed-files) to the Galaxy history
  - Import sample data to the Galaxy history
  - Upload only a single query track (bed-file) of interest, and evaluate the colocalization of the query against multiple reference tracks from a built-in collection
  - Use functionality from GSuite HyperBrowser to create track collections from files provided by the user or downloaded from online repositories following metadata search
- **Go to the coloc-stats tool**, to either
  - Use the basic mode to assess colocalization of your datasets according to the analytical approaches inherent in each selected tool
  - Use the advanced mode to assess colocalization of your datasets according to more narrowly specified analytical settings and assumptions
- **View your results, including**
  - Detailed results on colocalization for each combination of query and reference track
  - In case of multiple reference tracks, overview tables showing which reference tracks are most similar to the query according to the different tools

### Analysis example: colocalization of GATA1 with other chip-seq datasets

**Figure 2.**
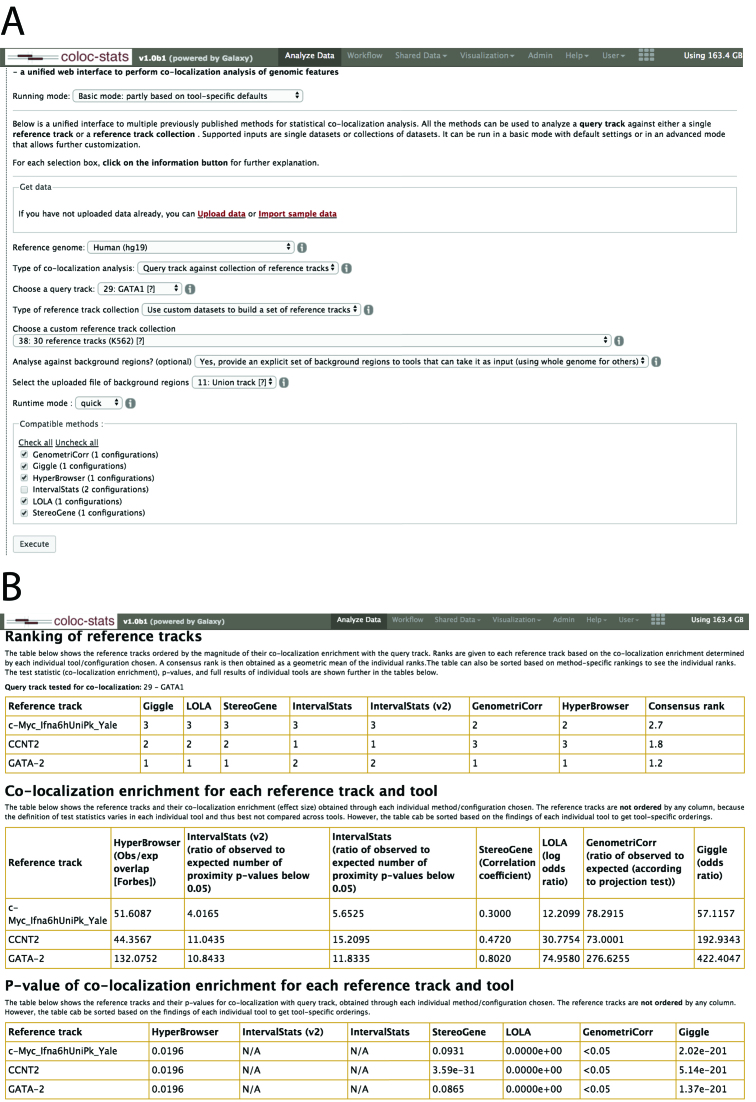
(**A**)The figure shows the Coloc-stats GUI in basic mode. Through selection lists and check boxes, the user can parameterize the tools according to analytical requirements. Each combination of selections results in a corresponding list of compatible tools, where there can be one or more compatible parameter configurations per tool. The user can then select a subset of tools to execute the analysis. (**B**)The figure shows a screenshot of the results page of a typical Coloc-stats analysis of one query track versus a collection of reference tracks. The results page is organized in four sections: (i) Ranking, where the reference tracks are ranked by the descriptive statistic according to each tool and parameter configuration, as well as a consensus column showing the averaged rank across methods; (ii) Descriptive statistics, one per reference track for each tool and parameter configuration is displayed; (iii) *P*-values, corresponding to each of the descriptive statistics (if the tool provides one) to assess the significance of each paired track analysis; (iv) Detailed results, list of links to the detailed results output of all tools for each reference track in the collection.

As an example scenario, we here consider the colocalization of different transcription factors (TFs). As input, we use a chip-seq peak dataset of GATA1 binding and assess the degree to which the dataset colocalizes with a collection of 30 other experimental datasets. Along with chip-seq peak datasets of other TFs, we also included chip-seq datasets for RNA polymerase II and a histone modification. We ran Coloc-stats with GATA1 as query track and the chip-seq collection as reference tracks, with each tool being run with its default options in basic mode (Figure 2A). The consensus rank showed a chip-seq track for a related TF GATA2 as the most highly colocalized, as well as showing high colocalization with other TFs suggested in the literature to have relations to GATA1, such as BRG1/SMARCA4 [(16,17)] (see Figure 2B). The lowest colocalization was consistently assigned to the trimethylation of lysine 27 on the H3 histone (H3K27me3), which is also very reasonable as this histone modification is associated with the formation of heterochromatic regions. The full analysis example is available at https://hyperbrowser.uio.no/coloc-stats/u/borissim/h/copy-of-gata-1-analysis-v1, where one in addition to the above described ranking can view enrichment scores, *P*-values and tool-specific output. A second example, on the analysis of colocalization of Schizophrenia lead single nucleotide polymorphism (SNPs) with DNaseII hypersensitivity regions, is also available at https://hyperbrowser.uio.no/coloc-stats/u/dianadom/h/coloc-stats-example-with-schizophrenia-snps.

## SYSTEM DESIGN AND IMPLEMENTATION

### Input data

Coloc-stats can be employed to perform colocalization analysis on any feature that can be represented as a set of coordinates on a reference genome. This encompasses a broad variety of genomic and epigenomic features of the genome that have been annotated in databases or that can be experimentally determined through the application of microarrays or high-throughput sequencing. Examples are datasets on exonic regions, DNA methylation state, histone modifications, experimental data on chromatin accessibility or association of variation at specific loci with traits or disease risk. The BED format has become a *de facto* standard for representing such data, and is used as input format in the Coloc-stats tool. Note that input files should adhere strictly to the to the file format specifications of the BED format [https://genome.ucsc.edu/FAQ/FAQformat.html#format1], where most importantly the first three fields need to be chromosome, start and end. For situations where users have datasets in other formats, the website includes a tool for converting from other formats like GFF or GTrack (tool:‘Convert between GTrack/BED/WIG/bedGraph/GFF/FASTA files’), as well as a tool for constructing a track file from nonstandard tabular data (tool:‘Create GTrack file from unstructured tabular data’).

For analyzing a query track against a collection, the standard Galaxy upload tool allows multiple tracks to be uploaded from local disk or from a list of URLs. Datasets of interest can then be combined in a single GSuite file (tool: ‘Create a GSuite from datasets in your history’), where this GSuite can be selected as input in the Coloc-stats tool. The web server also includes a selection of prebuilt collections of reference tracks that may be useful in a broad variety of settings, including collections generated by the authors of the LOLA and Giggle tools (9,13). These are here referred to as core databases, and can be selected directly in the Coloc-stats tool. At last, collections of tracks for specific epigenomic marks or relating to specific cell types can be easily constructed from datasets available in public repositories like ENCODE (18) or Roadmap Epigenomics (19) (tool:‘Create a GSuite from an integrated catalog of genomic datasets’).

Certain tools (4,6,9) accept a third track that defines which regions of the genome are to be included in the analysis. This track typically represents the universe of regions that could have possibly ended up in the genomic tracks of interest being queried for colocalization. As an example, when testing the colocalization of a set of disease-associated SNPs against other annotations, the background set could be all the SNPs covered by the technology platform, which are all assumed to have equal probability to be included in the SNP set of interest in the absence of any biological signal. As only a few tools accept such a third track, this track is not always required, and it is also possible to use a generic universe region set provided by Coloc-stats.

### Output

When a pairwise colocalization analysis (between two genomic tracks) is run through Coloc-stats, the output pages display a plot showing the negative logarithmic *P*-values obtained through different methods/configurations chosen. It also provides an initial assessment of the robustness of the findings. In addition, a table showing *P*-values, enrichment statistics and full results (as given out by the specific tool) of each tool/configuration is provided. If any chosen tool fails or reports an error, the error messages are shown in a separate table. When a query track is queried against a collection of reference tracks, the results page also shows an overview table where reference tracks are ordered by degree of colocalization with the query track, according to a consensus rank obtained through the multiple methods/configurations chosen.

### System architecture

The Coloc-stats web server is built upon a sophisticated infrastructure. Each tool is containerized as a Docker image (https://www.docker.com/) and wrapped by Common Workflow Language (https://www.commonwl.org/). The web interface is based on the Galaxy framework (20), which facilitates reproducible and transparent research by allowing users to easily share analysis histories as well as inspecting data and parameter selections underlying results of interest. The GUI selections are mapped to the lower-level architecture using Python. The web server is running in a virtual machine within a national cloud solution. We have made our codebase for integrating the various methods available for download as an easily installable package ‘pycolocstats’.

## CONCLUSION

Statistical assessment of colocalization often represents a final conclusive stage of an analysis, which entails many complications that if ignored can directly lead to erroneous conclusions in the form of false findings. It is thus advised to assess robustness of findings by employing alternative methodologies and carefully consider their underlying assumptions. We have thus developed Coloc-stats, which integrates a large number of published colocalization analysis tools, provides a unified user interface based on explicit selection of modeling assumptions and includes help pages and FAQs to guide on good scientific conduct for this setting. An easily accessible user interface allows multiple methods to be selected based on compatibility with analysis assumptions and run in parallel on datasets of interest.

## DATA AVAILABILITY

The Coloc-stats web server is freely and openly available at https://hyperbrowser.uio.no/coloc-stats/.
